# Perceived social support and academic life satisfaction among medical students: the mediating role of academic procrastination

**DOI:** 10.3389/fpsyg.2026.1888795

**Published:** 2026-07-30

**Authors:** Ayoob Lone, Aehsan Ahmad Dar, Arooj Arshad, Sayed Abdul Qadar Quadri, Nawaf Al Khashram, Maryam Jan, Reeman Haitham Battyor, Imtiyaz Ahmad Dar, Abdullah Abdulaziz Alnaim

**Affiliations:** 1 Department of Clinical Neurosciences, College of Medicine, King Faisal University, Al Ahsa, Saudi Arabia; 2 Department of Psychology, Akal University, Talwandi Sabo, Punjab, India; 3 Riphah Institute of Clinical & Professional Psychology, Riphah International University, Lahore, Pakistan; 4 Department of Biomedical Sciences, College of Medicine, King Faisal University, Al Ahsa, Saudi Arabia; 5 Medical Education Department, College of Medicine, King Faisal University, Al Ahsa, Saudi Arabia; 6 School Education Department, University of Kashmir, Srinagar, Jammu and Kashmir, India; 7 College of Medicine, King Faisal University, Al Ahsa, Saudi Arabia; 8 Department of Psychology, University of Kashmir, Srinagar, Jammu and Kashmir, India; 9 Department of Family and Community Medicine, College of Medicine, King Faisal University, Al Ahsa, Saudi Arabia

**Keywords:** academic life satisfaction, academic procrastination, mediation, medical students, perceived social support, PLS-SEM

## Abstract

**Background:**

Medical students experience substantial academic and demands that can adversely affect their academic wellbeing. Although perceived social support is a recognized protective factor, its relationship with academic life satisfaction and the mediating role of academic procrastination remain underexplored.

**Aim and objectives:**

The present study investigated the association between perceived social support and academic life satisfaction among medical students and examined the mediating role of academic procrastination.

**Methods:**

A quantitative cross-sectional study was conducted between February and April 2026 among 320 undergraduate medical students recruited through convenience sampling at a public university in AlAhsa, Saudi Arabia. Data were collected using the Multidimensional Scale of Perceived Social Support (MSPSS), Academic Procrastination Scale–Short Form (APS-SF), and Academic Life Satisfaction Scale. Data were analyzed using SPSS version 26 and SmartPLS3. Partial Least Squares Structural Equation Modeling (PLS-SEM) was used to evaluate the measurement and structural models and test the hypothesized mediation.

**Results:**

Participants reported moderate academic procrastination (*M* = 13.94, SD = 4.51), relatively high academic life satisfaction (*M* = 26.92, SD = 6.27), and high perceived social support (*M* = 5.15, SD = 1.27). Perceived social support was positively associated with academic life satisfaction (*β* = 0.431, *p* < 0.001) and negatively associated with academic procrastination (*β* = −0.156, *p* = 0.013). Academic procrastination was negatively associated with academic life satisfaction (*β* = −0.214, *p* < 0.001). Mediation analysis revealed a significant indirect effect of perceived social support on academic life satisfaction through academic procrastination (*β* = 0.033, *p* = 0.036), indicating complementary partial mediation.

**Conclusion:**

Perceived social support was positively associated with academic life satisfaction both directly and indirectly through reduced academic procrastination. Strengthening social support and reducing procrastination may enhance medical students’ academic well-being. These findings identify academic procrastination as a key behavioral mechanism linking social support with academic life satisfaction. However, the findings should be interpreted with caution because the cross-sectional design limits causal inference, and the single-center convenience sample may restrict the generalizability of the findings.

## Introduction

Medical education is widely recognized as one of the most psychologically demanding academic environments, characterized by persistent exposure to academic overload, competitive evaluation systems, and emotionally intensive training demands (Jafri et al., 2017). These demanding educational conditions often involve intensive curricula, high expectations, and continuous assessments, all of which place substantial psychological and academic pressure on students (Voltmer et al., 2021; Bergmann et al., 2019). Recent evidence from the Middle East indicates that psychological distress and academic stress are highly prevalent among medical students (Mahgoub et al., 2022; Abdel Aziz et al., 2023; Mohamed Hameed et al., 2026; Al-Huseini et al., 2026). In Saudi Arabia, studies have reported that medical students experience moderate-to-high academic stress, while a considerable proportion also report symptoms of psychological distress and burnout (Abozaid, 2024; Al-Shahrani et al., 2023; Jabali, 2025; Hassan et al., 2026; Tripathi et al., 2022). In addition, a significant proportion of medical students report burnout and emotional exhaustion, reflecting the demanding and prolonged nature of medical training (Al-Shahrani et al., 2023). These findings underscore the urgency of identifying protective psychosocial factors that can promote academic well-being and satisfaction among medical students.

Sustained exposure to academic stressors such as examinations, heavy workloads, and competitive learning environments can negatively affect students’ mental health and academic functioning (Bergmann et al., 2019; Jeyapalan and Blair, 2024). High levels of stress have been associated with reduced academic performance (Al-Roomy, 2026), impaired cognitive functioning (Dahlin et al., 2005), and reduced overall well-being (Cespedes-Londono et al., 2026). Given these challenges, it is essential to identify the factors that promote positive academic experiences and enhance student’s satisfaction with their academic experience.

Academic life satisfaction has emerged as a key outcome reflecting students’ overall evaluation of their academic experiences, including their sense of achievement, engagement, and fulfillment within the educational environment (Lent, 2004). In medical education, academic life satisfaction is particularly significant as it reflects student’s ability to adapt to rigorous academic demands while maintaining psychological well-being (Dyrbye et al., 2006). Higher academic life satisfaction has been associated with better academic adjustment, stronger motivation, and improved well-being, highlighting its importance as a key outcome in higher education research (Dyrbye et al., 2006; Balkis, 2013).

In recent years, increasing attention has been given to psychosocial and behavioral determinants that shape students’ academic well-being (Ruihua et al., 2025; Hassan et al., 2023; Petersen et al., 2023). Among psychosocial resources, perceived social support has been identified as a key protective factor. Based on Social Support Theory, social support refers to the perceived availability of emotional, informational, and instrumental assistance from significant others, including family, peers, and faculty (Cohen and Wills, 1985). In academic contexts, students who perceive strong social support generally report better emotional adjustment, greater academic engagement, and higher levels of academic satisfaction (Hassan et al., 2023; Shin and Park, 2022; Petersen et al., 2023). Beyond its direct influence on students’ academic experiences, perceived social support may also shape behavioral processes that facilitate or hinder academic functioning, particularly students’ ability to regulate their academic responsibilities.

Among the various behavioral factors that influence academic functioning, academic procrastination is particularly relevant in medical education because the curriculum requires sustained self-regulation, effective time management, and timely completion of multiple high-stakes academic and clinical responsibilities (Pereira et al., 2024). The demanding nature of medical training, characterized by intensive coursework, frequent examinations, and continuous performance expectations, may increase students’ vulnerability to procrastination, which can adversely affect both academic performance and psychological well-being (Procee et al., 2013; Araya-Castillo et al., 2023; Nazmifar et al., 2026).

At the behavioral level, academic procrastination represents a common maladaptive tendency among university students, particularly in demanding programs such as medicine. Academic procrastination is defined as the intentional delay of academic tasks despite expecting negative consequences (Ahmed et al., 2023). It is characterized by postponing essential academic activities such as studying or completing assignments even when deadlines are known (Bastani et al., 2018; Odacı et al., 2025). Previous research has shown that academic procrastination is associated with poor academic performances, and reduced psychological well-being (Martinie and Shankland, 2024; Ramadhani et al., 2026; Balkis, 2013).

Despite the recognized importance of both social support and academic procrastination in students’ academic experiences, relatively limited research has examined how these factors jointly relate to academic life satisfaction among medical students. Understanding these relationships may be particularly important within demanding medical education settings, where psychosocial resources and self-regulatory behaviors may substantially influence students’ academic well-being. Elucidating this mediation pathway has important practical implications for medical educators because it may help identify whether strengthening students’ social support networks can indirectly improve academic life satisfaction by reducing procrastination. Such evidence can inform the development of targeted interventions, including peer-support initiatives, faculty mentoring programs, and self-regulation training, aimed at promoting students’ academic success and psychological well-being throughout medical training.

### Theoretical framework

The present study is grounded in an integrated theoretical framework that combines Social Support Theory, Self-Regulation Theory, and Subjective Well-Being Theory to explain the relationships among perceived social support, academic procrastination, and academic life satisfaction among medical students. Together, these theoretical perspectives provide a comprehensive understanding of how interpersonal support and behavioral processes may shape students’ academic experiences and overall academic well-being within the demanding context of medical education.

Social Support Theory, proposed by Cohen and Wills (1985), suggests that social support functions as a protective psychosocial resource that helps individuals cope more effectively with stressful experiences by providing emotional reassurance, informational guidance, and practical assistance. In academic settings, students who perceive strong support from family members, peers, and significant others may feel more emotionally secure, academically confident, and better equipped to manage educational demands. Previous research has consistently shown that perceived social support is associated with better psychological adjustment, greater resilience, and more positive academic experiences among university students (Hassan et al., 2023; Shin and Park, 2022). Within medical education, where students are frequently exposed to intensive workloads, continuous assessments, and high academic expectations, supportive interpersonal relationships may play an especially important role in helping students adapt to academic stressors and maintain emotional well-being (Dyrbye et al., 2006; Ruihua et al., 2025).

The present study also draws upon Self-Regulation Theory to explain academic procrastination. According to Steel (2007), procrastination reflects difficulties in self-regulatory functioning, whereby individuals struggle to effectively manage motivation, emotions, and goal-directed behaviors. In academic environments, students who experience problems with self-regulation may postpone important academic tasks despite anticipating negative consequences. Existing evidence suggests that students who perceive stronger social support are less likely to engage in academic procrastination because supportive environments may enhance motivation, self-efficacy, and emotional regulation (Chen et al., 2025). Similarly, supportive social environments may strengthen students’ coping abilities, emotional stability, and academic confidence, thereby reducing tendencies toward procrastination (Yang et al., 2023; Miller et al., 2024). Conversely, students who perceive lower levels of social support may experience greater psychological strain and may be more likely to engage in avoidance-oriented coping behaviors such as academic procrastination (Handayani et al., 2021).

The study is further informed by Subjective Well-Being Theory, primarily developed by Diener (1984), which conceptualizes academic life satisfaction as an individual’s cognitive evaluation of their experiences, functioning, and overall quality of life. Within academic contexts, academic life satisfaction reflects students’ evaluative judgments regarding their academic experiences, achievements, and adjustment within the educational environment (Lent, 2004; Nogueira et al., 2019). Higher levels of academic life satisfaction have been associated with better academic adjustment, stronger motivation, and improved psychological well-being among university students (Dyrbye et al., 2006; Balkis, 2013). From this perspective, students’ academic well-being is shaped not only by academic performance, but also by interpersonal relationships, emotional experiences, and behavioral tendencies that influence how students perceive and respond to academic challenges.

Taken together, these theoretical perspectives suggest that perceived social support may be positively associated with academic life satisfaction both directly and indirectly through lower levels of academic procrastination. Students who perceive stronger social support may feel more capable of managing academic pressures, regulating their study behaviors, and remaining actively engaged with academic responsibilities. Reduced procrastination may subsequently contribute to lower stress, better academic adjustment, and more positive evaluations of academic experiences, ultimately leading to greater academic life satisfaction.

Existing empirical evidence generally supports these theoretical assumptions. Previous studies have linked perceived social support with improved academic adjustment, higher academic satisfaction, and better psychological well-being among university students (Hassan et al., 2023; Chen et al., 2025). Similarly, academic procrastination has consistently been associated with poorer academic functioning, increased stress, reduced productivity, and lower levels of academic satisfaction (Balkis, 2013; Demir and Kuşcu Karatepe, 2025; Rahmani et al., 2025). Building upon these theoretical and empirical foundations, the present study examines whether academic procrastination mediates the relationship between perceived social support and academic life satisfaction among medical students.

### Conceptual framework

**Figure 1:**
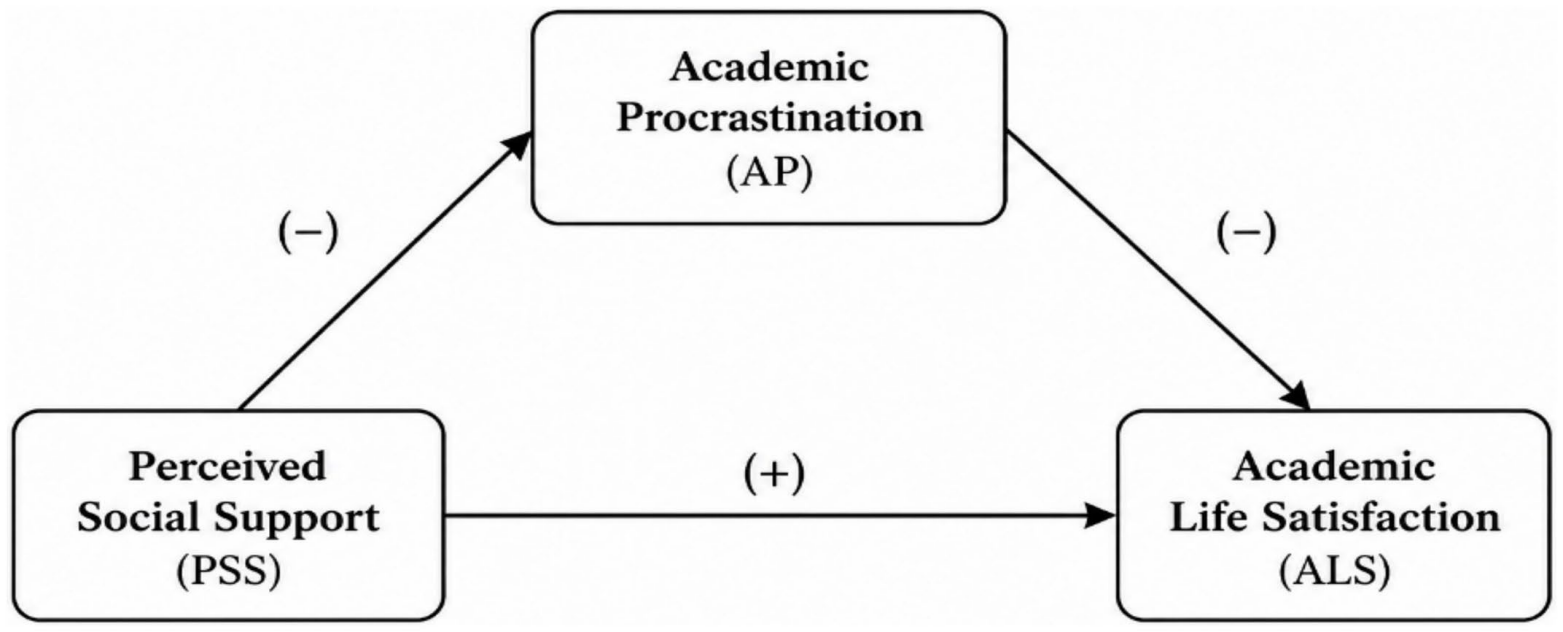
Hypothesized conceptual framework of the study.

Based on an integrated theoretical framework drawing from Social Support Theory, Self-Regulation Theory, and Subjective Well-Being Theory, the present study proposes that perceived social support is associated with academic life satisfaction both directly and indirectly through academic procrastination. Figure 1 illustrates the hypothesized conceptual framework of the study.

### Present study

Building upon the theoretical framework and empirical evidence presented above, the present study examined the relationships among perceived social support, academic procrastination, and academic life satisfaction among undergraduate medical students in Saudi Arabia. Specifically, the study investigated whether academic procrastination mediates the relationship between perceived social support and academic life satisfaction. By examining this mediation pathway, the study sought to provide evidence that may inform strategies to strengthen students’ psychosocial resources and self-regulatory behaviors, thereby promoting academic well-being in medical education. The following hypotheses were proposed:

*H1*: Perceived social support is positively associated with academic life satisfaction.

*H2*: Perceived social support is negatively associated with academic procrastination.

*H3*: Academic procrastination is negatively associated with academic life satisfaction.

*H4*: Academic procrastination mediates the relationship between perceived social support and academic life satisfaction.

## Methods and materials

### Study design and participants

This study employed a quantitative cross-sectional design to investigate the relationships among social support, academic procrastination, and academic life satisfaction among undergraduate medical students. Data were collected between February and April 2026 at a public university in the AlAhsa Governorate of Saudi Arabia. Participants were recruited using a convenience sampling approach during scheduled academic sessions and university visits conducted by the research team. A total of 356 eligible students were approached to participate in the study. Of these, 320 students completed the questionnaire, yielding a response rate of 89.9%. The remaining 36 students did not return completed questionnaires. All completed questionnaires were reviewed for completeness before data entry, and no questionnaires were excluded because of missing or incomplete responses. The study population consisted of undergraduate students enrolled in the Bachelor of Medicine and Bachelor of Surgery (MBBS) program. Eligible participants included Saudi MBBS students enrolled in First year to fifth year of the medical curriculum. Students from non-medical programs and those who did not provide informed consent were excluded from the study. Ethical approval was obtained from the Deanship of Scientific Research at King Faisal University (KFU-REC-2025-FEB-ETHICS3073). All study procedures were conducted in accordance with the ethical principles of the Declaration of Helsinki and established ethical guidelines for research involving human participants (World Medical Association, 2013).

### Sample size calculation

The required sample size was determined based on methodological recommendations for structural equation modeling (SEM), particularly Partial Least Squares Structural Equation Modeling (PLS-SEM). Previous literature suggests that SEM analyses generally require adequate sample sizes to ensure stable parameter estimation and sufficient statistical power, with recommended sample sizes commonly ranging between 200 and 400 participants for moderately complex mediation models (Kline, 2015; Hair et al., 2019). In addition, the final sample exceeded the minimum requirements recommended for PLS-SEM based on the “10-times rule” and statistical power considerations (Hair et al., 2019). Accordingly, the final sample of 320 undergraduate medical students was considered adequate for evaluating the proposed measurement and structural models and for testing the hypothesized mediation relationships.

### Measures

#### Academic procrastination scale–short form (APS-SF)

Academic procrastination was assessed using the APS-SF, originally developed by McCloskey (2011) and later psychometrically validated by Yockey (2016). The APS-SF is a unidimensional instrument designed to evaluate the tendency to delay academic tasks and responsibilities. The scale comprises five items rated on a 5-point Likert scale ranging from 1 (“strongly disagree”) to 5 (“strongly agree). Total scores range from 5 to 25, with higher scores indicating greater levels of academic procrastination. The Cronbach’s alpha value of the original scale was 0.87. In the present study, the APS-SF demonstrated good internal consistency reliability, with a Cronbach’s alpha coefficient of 0.79 among the study participants.

#### Academic life satisfaction scale

Academic life satisfaction was measured using the Academic Life Satisfaction Scale developed by Nogueira et al. (2019). The scale comprises 8 items and is structured into two sub-dimensions: personal satisfaction and satisfaction with the academic environment. Each item is rated on a 5-point Likert-type scale ranging from 1 (“strongly disagree”) to 5 (“strongly agree”), with higher scores indicating greater levels of academic life satisfaction. In the original validation study, the scale demonstrated acceptable internal consistency, with Cronbach’s alpha coefficients of 0.78 for the personal satisfaction sub-dimension and 0.73 for the satisfaction with academic environment sub-dimension. In the present study, the scale showed Cronbach’s alpha coefficients of 0.86, indicating excellent reliability for the current sample.

#### Perceived social support

Perceived social support was assessed using the Multidimensional Scale of Perceived Social Support (MSPSS), originally developed by Zimet et al. (1988). The MSPSS is a self-report instrument designed to evaluate individuals’ perceived support from three primary sources: family, friends, and significant others. The scale consists of 12 items distributed equally across the three sub-dimensions, with each dimension comprising four items. Participants responded to each item using a Likert-type scale ranging from 1 (“strongly disagree”) to 7 (“strongly agree”). Subscale scores were calculated by obtaining the mean score of the corresponding four items, while the overall perceived social support score was derived from the mean of all 12 items. Higher scores indicate greater perceived social support. Previous studies have reported high internal consistency reliability, with Cronbach’s alpha coefficients 0.93 (Adesola et al., 2025). In the present study, the MSPSS demonstrated excellent internal consistency reliability, with a Cronbach’s alpha coefficient of 0.94 for the overall scale.

#### Demographic information

A structured demographic information questionnaire was administered to obtain background characteristics of the participants. This questionnaire collected data related to personal, academic, and socio-economic variables, including participants’ age, sex, and educational status. In addition, information regarding family and living circumstances was obtained, such as place of residence, family system, monthly family income, and housing conditions. Collecting these variables provided a broader understanding of the socio-demographic profile of the study sample and allowed for contextual interpretation of the study findings.

### Procedure

Prior to the main data collection, a pilot study was conducted among 25 undergraduate medical students to evaluate the clarity, comprehensibility, and feasibility of the questionnaire. Feedback obtained from the pilot participants was used to make minor modifications to the wording and organization of the survey items to improve clarity and readability. The data obtained from the pilot study were not included in the final analysis. The questionnaire was administered in English, as all participants were proficient in English and routinely used it for their medical education. Following the pilot testing phase, the final questionnaire was distributed to eligible participants through personal contact by the researchers during on-site visits at the university. Before participation, students were informed about the objectives and procedures of the study, the voluntary nature of participation, and the confidentiality of their responses. Written informed consent was obtained from all participants prior to the commencement of data collection. Participants completed the self-administered questionnaire individually, and the average time required to complete the survey was approximately 10 min. Completed questionnaires were reviewed for completeness before inclusion in the final dataset.

### Statistical analysis

Data were analyzed using the Statistical Package for the Social Sciences (SPSS) version 26 for descriptive statistics and SmartPLS 3 software for Partial Least Squares Structural Equation Modeling (PLS-SEM). Descriptive statistics, including frequencies, percentages, means, standard deviations, and score ranges, were calculated to summarize participants’ demographic characteristics and the distribution of the study variables. The PLS-SEM approach was employed to test the hypothesized relationships among perceived social support, academic procrastination, and academic life satisfaction. PLS-SEM was considered appropriate because of its suitability for mediation analysis, prediction-oriented models, and the simultaneous assessment of measurement and structural models (Hair et al., 2019). The measurement model was first evaluated to establish the reliability and validity of the constructs. Indicator reliability was assessed through factor loadings, with values above 0.70 considered acceptable, although items with loadings above 0.40 were retained when composite reliability and convergent validity remained satisfactory (Hair et al., 2021). Internal consistency reliability was assessed using Cronbach’s alpha and composite reliability (CR), with values ≥0.70 indicating acceptable reliability (Büyüköztürk, 2019). Convergent validity was evaluated using the average variance extracted (AVE), where values above 0.50 indicated adequate convergent validity (Fornell and Larcker, 1981). Discriminant validity was examined using both the Fornell–Larcker criterion and the Heterotrait–Monotrait Ratio (HTMT). According to the Fornell–Larcker criterion, the square root of the AVE for each construct should exceed the inter-construct correlations (Fornell and Larcker, 1981). HTMT values below 0.90 were considered indicative of satisfactory discriminant validity (Henseler et al., 2015). Because all variables were collected using self-report measures within a single cross-sectional survey, common method variance was assessed using Harman’s single-factor test and the full collinearity variance inflation factor (VIF) approach proposed by Kock (2015). A first-factor variance below 50% and full collinearity VIF values below 3.3 were considered indicative that common method bias was unlikely to substantially affect the study findings. Following the assessment of the measurement model, the structural model was evaluated. Multicollinearity among predictor constructs was examined using inner Variance Inflation Factor (VIF) values, with values below 5 indicating the absence of multicollinearity (Shrestha, 2020). The explanatory power of the model was assessed using the coefficient of determination (*R*^2^). Path coefficients (*β*), *t*-values, and *p*-values and 95% bootstrap confidence intervals were estimated to test the proposed hypotheses. The significance of direct and indirect effects was assessed using the bootstrapping procedure with 5,000 resamples. Mediation analysis was conducted by examining the significance of the specific indirect effects following the recommendations of Zhao et al. (2010). As a robustness assessment, an additional structural model was estimated after adjusting for demographic and academic control variables (gender, academic year, GPA, area of residence, and daily time spent on social media).

## Results

### Sample characteristics

The demographic characteristics of the participants are presented in Table 1. A total of 320 undergraduate medical students participated in the study, with a slightly higher proportion of females than males. Participants were recruited from all academic years, with the highest representation from fourth-year students. The age distribution was relatively balanced across categories, with the largest proportion of students aged >23 years. Approximately three-quarters of the participants reported a GPA of 4.0 or above in the previous block. Regarding social media use, the majority of students reported spending between 3 and 4 h daily on social media, while only a small proportion used social media for less than 1 h per day. Most participants belonged to nuclear families, resided in urban areas, and reported a monthly family income above SAR 15,001. With respect to social media use during classroom sessions, the majority indicated using social media either sometimes or rarely. Additionally, most students were living with their families, whereas comparatively fewer students resided in university housing or shared apartments.

**Table 1:** Demographic characteristics of the study participants (*N* = 320).

Descriptive criteria		*N*	Percentages (%)
Gender	Male	151	47.2
	Female	169	52.8
Age	<20 years	114	35.6
	21–22 years	87	27.2
	>23 years	119	37.2
Academic year	Year 1	61	19.1
	Year 2	67	20.9
	Year 3	71	22.2
	Year 4	79	24.7
	Year 5	42	13.1
GPA in previous block	4.5–5	133	41.6
	4–4.4	103	32.2
	3–3.9	46	14.4
	<3	38	11.8
Time spent on social media	<1 h	9	2.8
	1–2 h	42	13.1
	2–3 h	70	21.9
	3–4 h	86	26.9
	4–5 h	58	18.1
	> 6 h	55	17.2
Family status	Nuclear	199	62.2
	Joint	121	37.8
Area of residence	Urban	222	69.4
	Rural	98	30.6
Family income	< 5,000 SAR	19	5.9
	5,001–10,000 SAR	70	21.9
	10,001–15,000 SAR	85	26.6
	> 15,001 SAR	146	45.6
Usage of social media during class	Never	46	14.4
	Rarely	98	30.6
	Sometimes	118	36.9
	Often	49	15.3
	Always	9	2.8
Type of stay	Living with family	263	82.2
	University Housing	40	12.5
	Sharing Apartment	17	5.3

The descriptive statistics of the study variables are presented in Table 2. The participants reported a moderate level of academic procrastination (Mean = 13.94, SD = 4.51). Academic life satisfaction was relatively high, with a mean score of 26.92 (SD = 6.27). Similarly, perceived social support was relatively elevated among participants, with a mean score of 5.15 (SD = 1.27). The observed score ranges were consistent with the possible ranges of each scale, indicating appropriate distribution of responses across all study variables.

**Table 2:** Descriptive statistics of the study variables.

Variable	Mean	*SD*	Actual score range	Possible score range
Academic procrastination	13.94	4.51	5–25	5–25
Academic life satisfaction	26.92	6.27	8–40	8–40
Perceived social support	5.15	1.27	1–7	1–7

Prior to evaluating the measurement model, common method variance was assessed because all variables were collected using self-report measures in a cross-sectional survey. Harman’s single-factor test indicated that the first unrotated factor explained 34.31% of the total variance, which is below the recommended threshold of 50%, suggesting that common method variance was unlikely to represent a serious concern. This conclusion was further supported by the full collinearity variance inflation factor (VIF) assessment. The full collinearity VIF values were 1.258 for Academic Procrastination, 1.018 for Academic Life Satisfaction, and 1.097 for Perceived Social Support, all of which were well below the recommended cut-off value of 3.3 (Kock, 2015). Collectively, these findings indicate that common method bias is unlikely to have materially influenced the study results.

### Measurement model

**Figure 2:**
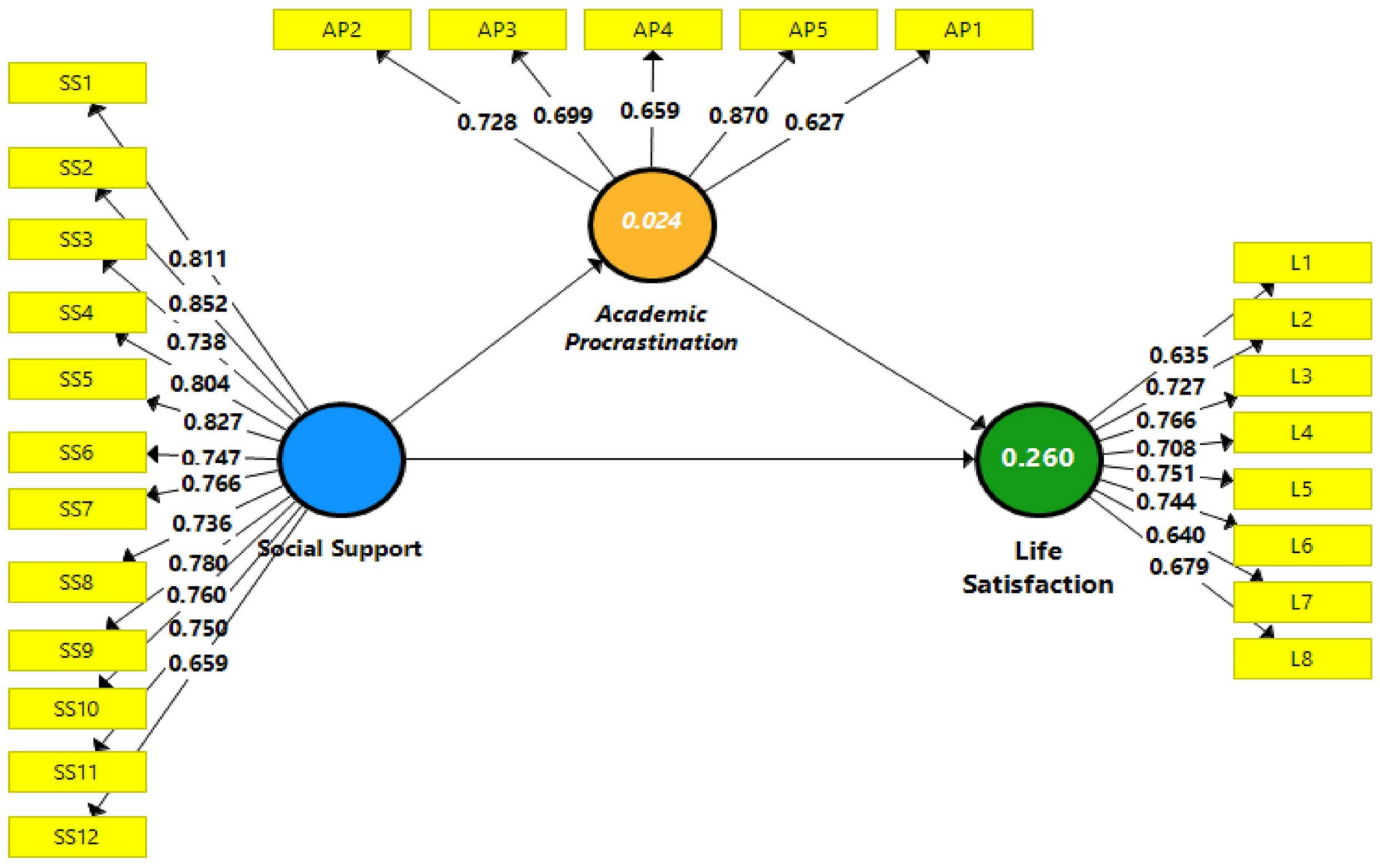
Measurement model.

To ensure the psychometric properties of the measurement model (Figure 2), the reliability and validity of the constructs were assessed. For this purpose, factor loadings, composite reliability (CR), convergent validity, and discriminant validity were examined, as presented in Tables 3–5. The results indicated that the majority of factor loadings exceeded the recommended threshold value of 0.70, confirming satisfactory indicator reliability (Hair et al., 2019). Although a few items (SS12, AP1, AP3, AP4, L1, L7, and L8) demonstrated factor loadings below 0.70 These indicators were retained to preserve construct content validity because their removal did not substantially improve construct reliability or convergent validity, and all constructs continued to demonstrate acceptable composite reliability and AVE values, consistent with recommended PLS-SEM guidelines (Hair et al., 2021).

**Table 3:** Factors loadings, reliability and validity of constructs.

Variables	Items	Loadings	Alpha	CR	AVE
Academic life satisfaction	LS1	0.635	0.859	0.889	0.501
	LS2	0.727			
	LS3	0.766			
	LS4	0.708			
	LS5	0.751			
	LS6	0.744			
	LS7	0.640			
	LS8	0.679			
Academic procrastination	AP1	0.627	0.789	0.843	0.521
	AP2	0.728			
	AP3	0.699			
	AP4	0.659			
	AP5	0.870			
Perceived social support	SS1	0.811	0.937	0.946	0.594
	SS2	0.852			
	SS3	0.738			
	SS4	0.804			
	SS5	0.827			
	SS6	0.747			
	SS7	0.766			
	SS8	0.736			
	SS9	0.780			
	SS10	0.760			
	SS11	0.750			
	SS12	0.659			

CR = Composite reliability; AVE = Average variance extracted.

**Table 4:** Discriminant validity (Fornell and Lacker).

	Variables	1	2	3
1	Academic procrastination	0.721		
2	Academic life satisfaction	−0.281	0.708	
3	Perceived social support	−0.156	0.464	0.771

**Table 5:** Heterotrait-Monotrait (HTMT) ratio for discriminant validity.

	Variables	1	2	3
1	Academic procrastination			
2	Academic life satisfaction	0.278		
3	Perceived social support	0.167	0.493	

The composite reliability values ranged from 0.843 to 0.946, exceeding the recommended threshold value of 0.70 and confirming strong internal consistency reliability (Hair et al., 2014). Furthermore, the average variance extracted (AVE) values ranged from 0.501 to 0.594, exceeding the minimum acceptable value of 0.50 and thereby confirming adequate convergent validity (Hair et al., 2010).

To evaluate the robustness of the measurement and structural models, a sensitivity analysis was performed after excluding indicators with outer loadings below 0.70 (AP1, AP3, AP4, L1, L7, L8, and SS12). Composite reliability changed only marginally (Academic Procrastination: 0.843 vs. 0.824; Academic Life Satisfaction: 0.889 vs. 0.868; Perceived Social Support: 0.946 vs. 0.945), whereas average variance extracted increased (Academic Procrastination: 0.521 vs. 0.703; Academic Life Satisfaction: 0.501 vs. 0.568; Perceived Social Support: 0.594 vs. 0.611). Re-estimation of the structural model resulted in only minor changes in the path coefficients and explained variance, and the substantive interpretation of the direct and indirect relationships remained unchanged. Accordingly, the original indicators were retained to preserve construct content validity.

Discriminant validity was assessed using the Fornell–Larcker criterion and the Heterotrait–Monotrait Ratio (HTMT). According to the Fornell–Larcker criterion, the square root of the AVE for each construct should be greater than its corresponding inter-construct correlations, thereby confirming that each construct is empirically distinct from the others (Fornell and Larcker, 1981). The results demonstrated that the square root of AVE for all constructs exceeded their respective inter-correlation values, indicating satisfactory discriminant validity (Table 4).

In addition, the HTMT approach was employed as a more rigorous assessment of discriminant validity. The results presented in Table 5 revealed that all HTMT values were below the recommended threshold value of 0.90, further confirming satisfactory discriminant validity among the constructs (Henseler et al., 2015).

### Structural model

The structural model assessment began with evaluating multicollinearity among the predictor constructs using inner Variance Inflation Factor (VIF) values. As shown in Table 6, all VIF values were below the recommended threshold of 5.0, indicating the absence of multicollinearity issues (Shrestha, 2020).

**Table 6:** Inner VIF values.

Predictor construct	Academic procrastination	Academic life satisfaction
Perceived social support	1.000	1.025
Academic procrastination		1.025

The explanatory power of the model was subsequently assessed using the coefficient of determination (R^2^). Path coefficients and their statistical significance were examined using the bootstrapping procedure in SmartPLS. As presented in Table 7, the model explained 2.4% of the variance in academic procrastination (*R*^2^ = 0.024) and 26.0% of the variance in academic life satisfaction (*R*^2^ = 0.260). According to Hair et al. (2019), the explanatory power for academic life satisfaction can be considered moderate, whereas the explanatory power for academic procrastination was weak. Although the explained variance for academic procrastination was relatively low, significant path coefficients may still indicate theoretically meaningful relationships despite modest predictive power (Hair et al., 2019). This finding also suggests that academic procrastination among medical students is likely influenced by multiple psychological, academic, and contextual factors beyond perceived social support alone.

**Table 7:** Coefficient of determination (*R*^2^).

Endogenous construct	*R* ^2^	Interpretation
Academic procrastination	0.024	Weak
Academic life satisfaction	0.260	Moderate

**Figure 3:**
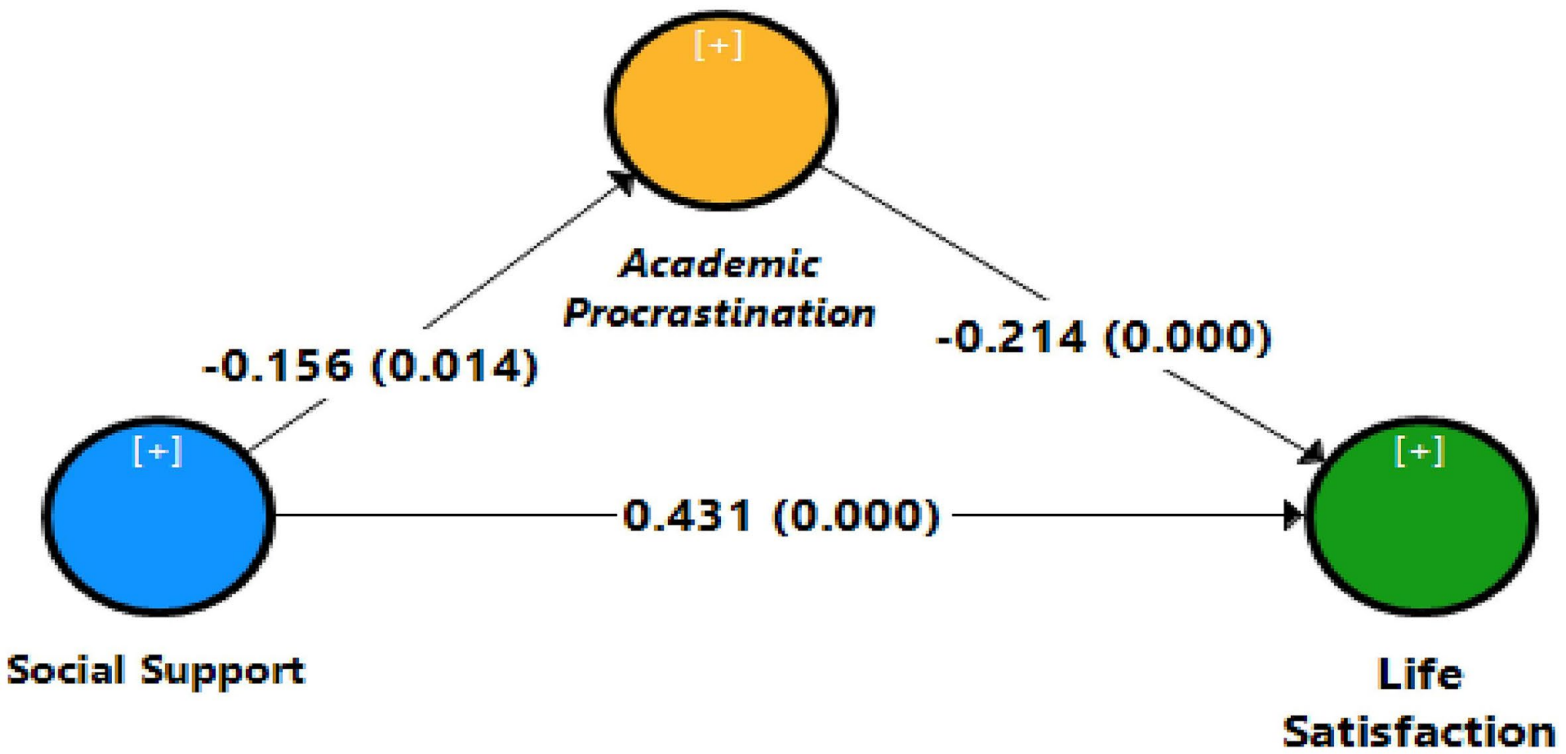
Structural model.

The hypothesized relationships were examined using the PLS-SEM approach in SmartPLS. Figure 3 illustrates the structural model, including the hypothesized relationships among the study constructs and their corresponding standardized path coefficients. The significance of the structural paths was assessed using the bootstrapping procedure with 5,000 resamples. The results of the structural model are presented in Table 8. As shown in Figure 3 and Table 8, perceived social support had a significant positive effect on academic life satisfaction (*β* = 0.431, *t* = 8.671, *p* < 0.001), whereas academic procrastination had a significant negative effect on academic life satisfaction (*β* = −0.214, *t* = 3.859, *p* < 0.001). Therefore, hypotheses H1 and H3 were supported. In addition, perceived social support demonstrated a significant negative association with academic procrastination (*β* = −0.156, *t* = 2.483, *p* = 0.013), thereby supporting H2. Furthermore, the mediating role of academic procrastination in the relationship between perceived social support and academic life satisfaction was examined through the assessment of specific indirect effects following the recommendations of Zhao et al. (2010). The results indicated that the indirect effect of perceived social support on academic life satisfaction through academic procrastination was statistically significant (*β* = 0.033, *t* = 2.094, *p* = 0.036), thereby supporting H4. However, the magnitude of the indirect effect was small, indicating that academic procrastination accounted for only a limited proportion of the observed association between perceived social support and academic life satisfaction. Because both the direct and indirect effects were statistically significant and operated in the same direction, the findings indicate the presence of complementary partial mediation. This suggests that perceived social support enhances academic life satisfaction both directly and indirectly through reducing academic procrastination.

**Table 8:** Standardized path coefficients and statistical significance.

Hypotheses	Hypothesized path	*β*	*T*-value	*p*-values	Decision
H1	Perceived social support → Academic life satisfaction	0.431	8.671	< 0.001	Supported
H2	Perceived social support → Academic procrastination	−0.156	2.483	0.013	Supported
H3	Academic procrastination → Academic life satisfaction	−0.214	3.859	< 0.001	Supported
H4	Perceived social support → Academic procrastination → Academic life satisfaction	0.033	2.094	0.036	Supported

To examine the robustness of the proposed structural model, an additional PLS-SEM analysis was conducted after adjusting for gender, academic year, grade point average (GPA), area of residence, and daily time spent on social media. As presented in Table 9, the principal structural relationships remained stable following adjustment. Perceived social support remained negatively associated with academic procrastination (*β* = −0.156, 95% CI [−0.277, −0.054]), academic procrastination remained negatively associated with academic life satisfaction (*β* = −0.189, 95% CI [−0.303, −0.085]), and perceived social support remained positively associated with academic life satisfaction (*β* = 0.448, 95% CI [0.349, 0.548]). Among the control variables, only GPA demonstrated a significant association with academic life satisfaction (*β* = −0.117, 95% CI [−0.220, −0.013]), whereas gender, academic year, area of residence, and daily time spent on social media were not significantly associated with academic life satisfaction. The indirect effect of perceived social support on academic life satisfaction through academic procrastination remained small (*β* = 0.029), and the 95% bias-corrected bootstrap confidence interval excluded zero (95% BCa CI [0.002, 0.059]). Collectively, these findings indicate that the substantive interpretation of the proposed model remained robust after adjustment for demographic and academic characteristics.

**Table 9:** Robustness analysis after adjustment for demographic and academic control variables.

Structural path	*β*	95% bootstrap confidence interval	Interpretation
Perceived social support → Academic procrastination	−0.156	[−0.277, −0.054]	Significant
Academic procrastination → Academic life satisfaction	−0.189	[−0.303, −0.085]	Significant
Perceived social support → Academic life satisfaction	0.448	[0.349, 0.548]	Significant
GPA → Academic life satisfaction	−0.117	[−0.220, −0.013]	Significant
Gender → Academic life satisfaction	−0.093	[−0.311, 0.123]	Not significant
Academic year → Academic life satisfaction	−0.019	[−0.124, 0.084]	Not significant
Area of residence → Academic life satisfaction	−0.103	[−0.318, 0.113]	Not significant
Time spent on social media → Academic life satisfaction	−0.079	[−0.180, 0.015]	Not significant
Perceived social support → Academic procrastination → Academic life satisfaction	0.029	95% BCa CI [0.002, 0.059]	Significant

β = standardized path coefficient; CI = 95% bias-corrected and accelerated (BCa) bootstrap confidence interval obtained from 5,000 bootstrap resamples. Confidence intervals that do not include zero indicate evidence of an effect.

## Discussion

The present study examined the relationships among perceived social support, academic procrastination, and academic life satisfaction among undergraduate medical students using a PLS-SEM approach. Overall, the findings demonstrated that perceived social support was positively associated with academic life satisfaction both directly and indirectly through lower levels of academic procrastination. Notably, the direct association between perceived social support and academic life satisfaction was substantially stronger than the indirect pathway through academic procrastination, indicating that supportive interpersonal relationships primarily promote students’ academic well-being through direct emotional and psychosocial benefits, with reduced procrastination serving as a complementary behavioral mechanism. These findings highlight the importance of supportive interpersonal relationships in promoting academic well-being and adjustment within demanding medical education environments.

The findings indicated that perceived social support was positively associated with academic life satisfaction, thereby supporting H1. The positive association between perceived social support and academic life satisfaction suggests that perceived social support may function as an important psychological resource that enhances students’ academic adjustment and emotional wellbeing. This finding is consistent with previous research showing that social support is associated with better psychological adjustment, greater resilience, and improved academic wellbeing among university students (Petersen et al., 2023; Shin and Park, 2022; Ruihua et al., 2025; Hassan et al., 2023). Students who perceive stronger emotional, informational, and interpersonal support from family members, peers, and significant others may feel more emotionally secure, valued, and capable of coping with academic demands. Such support may strengthen students’ sense of belonging and emotional security, thereby contributing to more positive academic experiences and higher levels of academic life satisfaction. Within medical education settings, where students are frequently exposed to substantial academic pressure and emotionally demanding training environments, social support may function as an important psychosocial resource that facilitates academic adaptation and emotional well-being (Dyrbye et al., 2006). In collectivist cultural contexts such as Saudi Arabia, interpersonal relationships and family support often carry substantial emotional and social significance, which may further strengthen the association between perceived social support and students’ academic experiences and wellbeing.

The study also found that perceived social support was negatively associated with academic procrastination, supporting H2. Students who perceived stronger social support were less likely to report procrastination behaviors. These results align with previous literature suggesting that supportive social environments are associated with lower tendencies toward academic procrastination (Chen et al., 2025; Miller et al., 2024; Yang et al., 2023; Handayani et al., 2021). One possible explanation is that perceived social support may provide emotional encouragement, practical guidance, and academic reassurance, helping students manage academic stress more effectively and maintain greater confidence in handling academic responsibilities. Supportive interpersonal relationships may also foster accountability, motivation, and adaptive self-regulation, thereby reducing tendencies toward delaying academic tasks. Within demanding medical education environments, where students are frequently exposed to heavy workloads, continuous assessments, and examination-related stress, strong support systems may help students remain academically engaged and psychologically resilient. Nevertheless, the relatively low explained variance observed for academic procrastination suggests that procrastination among medical students is likely shaped by multiple interacting psychological and contextual influences beyond perceived social support alone. Academic procrastination may therefore reflect a broader combination of academic pressures, emotional challenges, and self-regulatory difficulties commonly experienced within medical education settings (Voltmer et al., 2021; Bergmann et al., 2019).

The findings further demonstrated that academic procrastination was negatively associated with academic life satisfaction, thereby supporting H3. Students who reported higher levels of academic procrastination tended to experience lower satisfaction with their academic life. These results align with previous studies linking procrastination with poorer academic adjustment, increased stress, and lower levels of academic life satisfaction among university students (Demir and Kuşcu Karatepe, 2025; Rahmani et al., 2025; Balkis, 2013). One possible interpretation is that persistent procrastination may generate feelings of guilt, academic inefficiency, and reduced perceived control over academic responsibilities, which may negatively shape students’ evaluations of their academic experiences. Delaying academic tasks may also increase academic burden over time and contribute to feelings of academic pressure and dissatisfaction. In rigorous medical education environments, persistent procrastination may interfere with effective learning, examination preparation, and overall academic adjustment, thereby negatively influencing students’ academic wellbeing.

Importantly, the mediation analysis demonstrated that academic procrastination significantly mediated the relationship between perceived social support and academic life satisfaction, thereby supporting H4. Specifically, higher perceived social support was associated with lower levels of academic procrastination, which in turn was associated with greater academic life satisfaction. This mediation pathway may be understood through the perspectives of Social Support Theory and Self-Regulation Theory. Social Support Theory proposes that supportive interpersonal relationships provide emotional reassurance, informational guidance, and practical assistance that help individuals cope more effectively with stressful experiences (Cohen and Wills, 1985). Within demanding medical education environments, these supportive resources may reduce academic stress and strengthen students’ confidence in managing academic responsibilities. From the perspective of Self-Regulation Theory, effective regulation of thoughts, emotions, and goal-directed behaviors enables students to manage academic demands and complete tasks in a timely manner, whereas difficulties in self-regulation increase susceptibility to procrastination (Steel, 2007). Accordingly, students who perceive stronger social support may be better equipped to regulate their academic behaviors, thereby reducing procrastination and promoting greater academic life satisfaction.

The present findings are also consistent with previous studies showing that students with higher levels of perceived social support tend to report lower academic procrastination because supportive environments may enhance motivation, self-efficacy, and emotional regulation (Chen et al., 2025). Prior research has additionally shown that lower levels of procrastination are associated with better academic adjustment, greater satisfaction, and improved psychological wellbeing among university students (Demir and Kuşcu Karatepe, 2025; Rahmani et al., 2025). However, although the indirect effect was statistically significant, its magnitude was small, indicating that academic procrastination accounted for only a limited proportion of the observed association between perceived social support and academic life satisfaction. Accordingly, the association between perceived social support and academic life satisfaction was reflected predominantly in the direct pathway, whereas the indirect pathway through academic procrastination contributed only a limited proportion of the overall association. These findings suggest that academic procrastination represents only one of several potential behavioral and psychological mechanisms through which perceived social support is associated with academic life satisfaction. Future research should therefore examine additional mechanisms, such as academic self-efficacy, resilience, coping strategies, or psychological wellbeing, that may further account for this relationship.

### Limitations and future directions

Several limitations of this study should be acknowledged. First, the cross-sectional design limits the ability to infer causal relationships among perceived social support, academic procrastination, and academic life satisfaction. Therefore, the observed associations should be interpreted as correlational rather than causal, and longitudinal studies are recommended to better establish temporal and directional relationships among the study variables. Second, the study relied on self-reported questionnaires, which may be subject to response biases, including recall bias and social desirability bias. Because all variables were measured using self-report instruments at a single time point, common method variance cannot be completely ruled out. However, Harman’s single-factor test and full collinearity VIF assessments indicated that common method bias was unlikely to have materially influenced the observed relationships. Nevertheless, future studies should consider using longitudinal, multi-source, or objective measures to further minimize potential method-related bias and strengthen causal inference. Third, although the participants represented students from different regions of Saudi Arabia, the study was conducted within a single medical college, which may limit the broader generalizability of the findings to students from other universities, academic disciplines, and cultural settings. Medical students may experience unique academic pressures and psychological demands that differ from those experienced by students in other academic programs. Fourth, perceived social support was examined as a higher-order latent construct rather than as its individual dimensions (i.e., family, friends, and significant others). Although this approach was appropriate for testing the proposed theoretical model, it did not allow the independent contributions of different sources of social support to academic procrastination and academic life satisfaction to be examined. Given the importance of family relationships within the Saudi Arabian collectivist context, future research should formulate more granular hypotheses and investigate whether family, peer, and significant-other support differentially influence medical students’ self-regulation and academic well-being. Finally, while academic procrastination was examined as a mediating variable, other potentially important psychological and contextual factors—such as academic stress, self-efficacy, coping strategies, resilience, burnout, and personality characteristics—were not included in the model. Future research should incorporate these variables to provide a more comprehensive understanding of factors associated with academic life satisfaction among university students.

### Implications

The findings of this study contribute to the existing literature by integrating Social Support Theory, Self-Regulation Theory, and Subjective Well-Being Theory to explain academic life satisfaction among medical students. The results suggest that academic procrastination may represent an important behavioral pathway through which perceived social support is associated with academic well-being. In doing so, the study extends previous research by highlighting the combined influence of interpersonal and behavioral factors in shaping students’ academic experiences and perceptions of academic satisfaction.

From a practical perspective, the findings highlight the importance of strengthening supportive academic environments within medical education settings. Universities may benefit from encouraging positive relationships among students, peers, and faculty members through structured mentorship programs, peer-support initiatives, and accessible academic counselling services. Within medical education, these support systems may be integrated into preclinical and clinical training by establishing faculty mentoring during clinical rotations, peer-support groups, and regular academic advising to help students cope with demanding coursework, clinical responsibilities, and emotionally challenging learning experiences. Such interventions may be particularly valuable in medical education environments, where sustained academic pressure and competitive learning conditions can negatively affect students’ emotional well-being and academic engagement. In addition, interventions targeting academic procrastination—such as time-management training, self-regulation workshops, and motivational enhancement programs—may help students manage academic responsibilities more effectively and improve their overall academic experiences. Furthermore, self-regulation interventions should be tailored to the unique demands of medical training by incorporating time-management, study-planning, and goal-setting strategies that assist students in balancing intensive curricula, clinical placements, and preparation for high-stakes professional examinations. Overall, the findings underscore the importance of strengthening supportive academic environments and addressing maladaptive academic behaviors to enhance academic well-being among medical students.

## Conclusion

This study examined the relationship between perceived social support and academic life satisfaction among undergraduate medical students, with academic procrastination evaluated as a mediating mechanism. The findings demonstrated that perceived social support was positively associated with academic life satisfaction, whereas academic procrastination was negatively associated with academic life satisfaction. In addition, students who reported stronger perceived social support were less likely to engage in academic procrastination. Importantly, academic procrastination partially mediated the relationship between perceived social support and academic life satisfaction, suggesting that supportive interpersonal environments may contribute to students’ academic wellbeing both directly and indirectly through improved self-regulatory functioning. These findings highlight the combined importance of interpersonal and behavioral factors in shaping students’ academic experiences within demanding medical education settings. Overall, the study contributes to the growing literature on academic well-being by identifying academic procrastination as one behavioral pathway linking perceived social support with academic life satisfaction among medical students. Strengthening supportive academic environments and implementing interventions aimed at reducing academic procrastination may therefore represent valuable strategies for enhancing student well-being and academic adjustment in medical education contexts.

## Data Availability

The raw data supporting the conclusions of this article will be made available by the authors, without undue reservation.
